# Role of iRhoms 1 and 2 in Endochondral Ossification

**DOI:** 10.3390/ijms21228732

**Published:** 2020-11-19

**Authors:** Renpeng Fang, Coline Haxaire, Miguel Otero, Samantha Lessard, Gisela Weskamp, David R. McIlwain, Tak W. Mak, Stefan F. Lichtenthaler, Carl P. Blobel

**Affiliations:** 1 Department of Orthopaedics, Xiangya Hospital, Central South University, Changsha 410008, China; fangr@hss.edu; 2 Arthritis and Tissue Degeneration Program, Hospital for Special Surgery at Weill Cornell Medicine, New York, NY 10021, USA; Coline.haxaire@gmail.com (C.H.); weskampg@hss.edu (G.W.); 3 Orthopedic Soft Tissue Research Program, Hospital for Special Surgery at Weill Cornell Medicine, New York, NY 10021, USA; OteroM@HSS.EDU (M.O.); lessards@hss.edu (S.L.); 4 Baxter Laboratory in Stem Cell Biology, Department of Pathology, Stanford University School of Medicine, Stanford, CA 94305, USA; mcilwain@stanford.edu; 5 Campbell Family Institute for Breast Cancer Research, Ontario Cancer Institute, University Health Network, Toronto, ON M5G 2M9, Canada; tak.mak@uhnresearch.ca; 6 German Center for Neurodegenerative Diseases (DZNE), 81377 Munich, Germany; stefan.lichtenthaler@tum.de; 7 Neuroproteomics, School of Medicine, Klinikum rechts der Isar, Technical University of Munich, 81675 Munich, Germany; 8 Munich Cluster for Systems Neurology (SyNergy), 81377 Munich, Germany; 9 Institute for Advanced Study, Technische Universität München, 85748 Garching, Germany; 10 Department of Medicine, Department of Biophysics, Physiology and Systems Biology, Weill Cornell Medicine, New York, NY 10021, USA

**Keywords:** a disintegrin and metalloprotease 17, ADAM17, inactive Rhomboid 1, 2, iRhom1, 2, endochondral ossification

## Abstract

Growth of the axial and appendicular skeleton depends on endochondral ossification, which is controlled by tightly regulated cell–cell interactions in the developing growth plates. Previous studies have uncovered an important role of a disintegrin and metalloprotease 17 (ADAM17) in the normal development of the mineralized zone of hypertrophic chondrocytes during endochondral ossification. ADAM17 regulates EGF-receptor signaling by cleaving EGFR-ligands such as TGFα from their membrane-anchored precursor. The activity of ADAM17 is controlled by two regulatory binding partners, the inactive Rhomboids 1 and 2 (iRhom1, 2), raising questions about their role in endochondral ossification. To address this question, we generated mice lacking iRhom2 (*iR2−/−*) with floxed alleles of iRhom1 that were specifically deleted in chondrocytes by Col2a1-Cre (*iR1∆Ch*). The resulting *iR2−/−iR1∆Ch* mice had retarded bone growth compared to *iR2−/−* mice, caused by a significantly expanded zone of hypertrophic mineralizing chondrocytes in the growth plate. Primary *iR2−/−iR1∆Ch* chondrocytes had strongly reduced shedding of TGFα and other ADAM17-dependent EGFR-ligands. The enlarged zone of mineralized hypertrophic chondrocytes in *iR2−/−iR1∆Ch* mice closely resembled the abnormal growth plate in *A17∆Ch* mice and was similar to growth plates in *Tgfα−/−* mice or mice with EGFR mutations. These data support a model in which iRhom1 and 2 regulate bone growth by controlling the ADAM17/TGFα/EGFR signaling axis during endochondral ossification.

## 1. Introduction

The appendicular and axial skeleton develops through a process referred to as endochondral ossification, in which a highly-specialized cartilage intermediate represents an essential part of bone growth and maturation [1,2,3]. During bone growth through endochondral ossification, three different zones of chondrocytes are present in the growth plate, which are referred to as the resting zone, the proliferating zone, and the zone of hypertrophic chondrocytes [2,3,4]. At the boundary to the chondro-osseous junction, the zone of hypertrophic chondrocytes ends in a layer of cells that are terminally differentiated and become mineralized before they are thought to undergo apoptosis and remodel into trabecular bone. This is accompanied by an invasion of highly specialized endothelial cells as well as an influx of bone eroding osteoclasts and bone synthesizing osteoblasts. 

Previous studies on the signaling pathways involved in regulating the function of hypertrophic chondrocytes have uncovered an important role of a disintegrin and metalloprotease 17 (ADAM17) and epidermal growth factor receptor (EGFR) signaling in this process. ADAM17 is a cell surface metalloprotease that is required for EGFR signaling during mouse development. ADAM17 has an essential role in liberating several ligands of the EGFR from their membrane anchor (transforming growth factor a, TGFα; heparin binding epidermal growth factor-like growth factor, HB-EGF; epiregulin, EREG; amphiregulin, AREG; and epigen, EPGN), which in turn is crucial for their functional activation [5,6]. Therefore, mice lacking ADAM17 resemble mice lacking the EGFR or specific EGFR ligands [7,8,9,10,11,12]. *Adam17−/−* mice die shortly after birth with open eyes, caused by lack of TGFα/HB-EGF signaling that is required for prenatal eyelid closure in mice, which are born with closed eyes [8,13]. *Adam17−/−* mice also have enlarged and misshapen heart valves, caused by a lack of HB-EGF signaling that results in increased smad1/5/8 and BMP signaling and abnormal morphogenesis of the endocardial cushion [14]. Finally, *Adam17−/−* mice have an enlarged zone of mineralized hypertrophic cells in their growth plate, most likely caused by defects in TGFα signaling [15,16,17]. These findings are consistent with the known role of the EGFR in regulating endochondral ossification and differentiation of hypertrophic chondrocytes [18,19,20]. Knock-in mice with the human EGFR have reduced EGFR signaling [21], resulting in a hypomorphic phenotype with an enlarged zone of hypertrophic cells in the growth plate. This phenotype is also seen in developing rats treated with the EGFR-inhibitor Gefitinib or in mice lacking the EGFR in chondrocytes [19], but not in mice lacking the EGFR in osteoclasts [22], suggesting that the EGFR signaling in chondrocytes is crucial for normal endochondral ossification [20]. The enlarged growth plate is thought to be caused, at least in part, through reduction in matrix metalloproteinase 13 (MMP13) signaling, which, together with MMP14 and MMP9, has been implicated in regulating chondrocyte maturation in the growth plate (reviewed in [23]). Moreover, the EGFR has important roles in osteoblast differentiation and anabolic bone metabolism [22,24,25]. Taken together, previous studies have demonstrated that chondrocyte proliferation and differentiation is regulated by the ADAM17/EGFR signaling pathway, with TGFα functioning as the best candidate EGFR-ligand responsible for regulating these processes [17,19].

The goal of this study was to explore the role of the recently identified regulators of ADAM17, the seven-membrane-spanning inactive Rhomboid proteins iRhom1 and 2, in bone growth and endochondral ossification. iRhom2 was initially discovered as a critical regulator of the maturation and function of ADAM17 in myeloid cells, where one of its well-characterized substrates is the pro-inflammatory cytokine TNFα [26,27]. Further studies showed that iRhom2 controls the substrate selectivity of ADAM17, as evidenced by the lack of stimulated release of several cell surface proteins that are cleaved by ADAM17, including HB-EGF and Epiregulin [28]. However, stimulated shedding of the EGFR-ligand TGFα can be supported by both iRhom2 and the related iRhom1 [28], raising questions about the role of the two iRhoms in endochondral bone formation. Further studies showed that iRhom2 and the related iRhom1 have largely overlapping expression in mice, although iRhom2 is not expressed in the brain (except in microglia), whereas iRhom1 is not expressed in most immune cells, including myeloid cells [29,30]. Two groups generated mice, in which both iRhom1 and 2 were simultaneously inactivated, resulting in perinatal or embryonic lethality [30,31]. In one study, *iRhom1/2−/−* double knockout mice died perinatally with open eyes at birth, defective heart valves and enlarged growth plates [30], similar to mice lacking ADAM17 [8]. The perinatal lethality of *iRhom1/2* double knockout mice prevented an analysis of the role of these two iRhoms in bone development after birth. To explore how the lack of iRhom1 and 2 affects endochondral ossification during postnatal bone growth, we generated mice that lacked iRhom2 systemically and also carried floxed alleles of iRhom1, which were specifically deleted in chondrocytes through the Col2a-Cre transgene. In these animals, we would expect a loss of mature ADAM17 in chondrocytes, as these cells should lack both iRhoms. Moreover, we would expect that ADAM17 is inactivated in myeloid cells through the systemic lack of iRhom2 [26,27,29]. Importantly, the inactivation of iRhom2 would also restrict the substrate selectivity of ADAM17 for proteins such as HB-EGF in other cells in the body that still express iRhom1, which could conceivably result in a different growth plate or bone phenotype compared to *Adam17∆Ch* mice. Here, we report that the *iRhom2−/−iRhom1fl/fl-Col2a1-Cre* (referred to as *iR2−/−iR1∆Ch*) mice have an enlarged zone of hypertrophic cells and closely resemble *Adam17∆Ch* mice, which demonstrates that iRhom1 and iRhom2 are key regulators of the function of ADAM17 in chondrocytes during endochondral ossification in mice.

## 2. Results

### 2.1. Expansion of the Zone of Hypertrophic Chondrocytes in the Femur Growth Plate of iR2−/−iR1∆Ch Mice

The main goal of this study was to learn more about the contribution of iRhom1 and iRhom2 to endochondral ossification. Since mice lacking both iRhom1 and 2 systemically die shortly after birth, similar to *Adam17−/−* mice [30], yet mice lacking either iRhom1 or iRhom2 alone appear normal and healthy [26,27,30], we generated *iRhom2−/−* mice carrying two floxed alleles of iRhom1 and the chondrocyte selective Col2a1-Cre [15,32]. These *iRhom2−/−iRhom1fl/fl/Col2a1-cre* mice (referred to as *iR2−/−iR1∆Ch* mice) were expected to lack both iRhoms in chondrocytes, but to express only iRhom1, not iRhom2 in all other cells and tissues where Col2a1-Cre is not expressed. In matings of *iR2−/−iR1∆Ch* with *iR2−/−iR1fl/fl* animals that lacked the Col2a1-Cre, the resulting *iR2−/−iR1∆Ch* offspring were born at the expected Mendelian ratio (total number of mice, 118, *iR2−/−iR1∆Ch*: 55 (47%), *iR2−/−iR1fl/fl*: 63 (53%)) and were viable and fertile with no evident spontaneous pathological phenotypes or abnormalities during routine handling.

**Figure 1:**
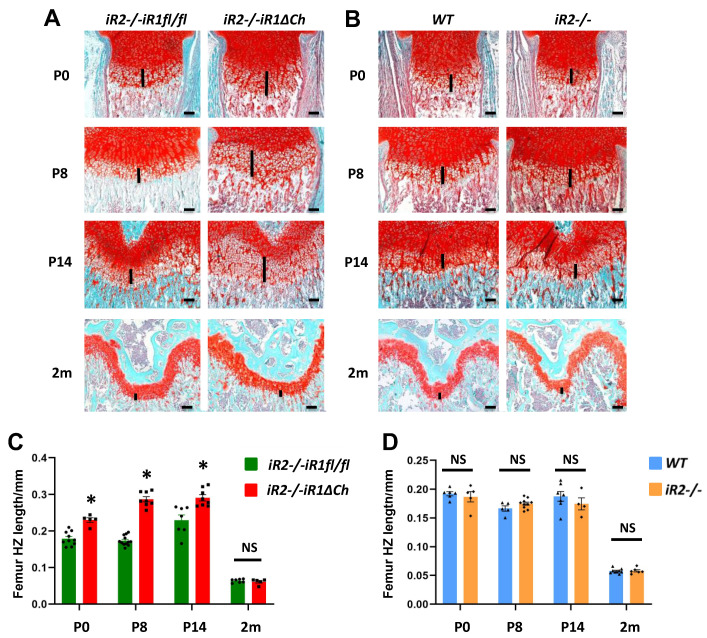
The zone of hypertrophic chondrocytes is enlarged in *iR2−/−iR1∆Ch* mice compared to controls. (**A**,**B**) Sections of the distal femoral growth plate from *iR2−/−iR1fl/fl* controls or *iR2−/−iR1∆Ch* mutants (**A**) or from wild type (WT) or *iR2−/−* mice (**B**) at different ages (postnatal day P0, P8, P14 and 2 months (m) of age) were stained with Safranin O and Fast Green to highlight bone in light blue and cartilage and the chondrocytes in the growth plate in red. The length of the zone of hypertrophic cells is indicated by a black line in each panel. (**C**,**D**) Quantification of the results shown in panels A and B shows an increase in the length of the zone of hypertrophic cells in *iR2−/−iR1∆Ch* samples compared to *iR2−/−iR1fl/fl* controls at P0, P8 and P14, but not at 2 months (**C**), whereas there was no significant difference between WT and *iR2−/−* samples at any of these four time points (**D**). The scale bar represents 100 µm in each panel. * indicates statistical significance with a *p*-value of < 0.05 in a student’s t-test, whereas NS indicates no significant change. The quantification was performed on at least 4 samples from separate animals per day and genotype (for WT P0, *n* = 6; P8, *n* = 5; P14, *n* = 7; 2m, *n* = 9; for *iR2−/−* P0, *n* = 5; P8, *n* = 10; P14, *n* = 4; 2m, *n* = 6; for *iR2−/−iR1fl/fl*, P0, *n* = 10; P8, *n* = 12; P14, *n* = 7; 2m, *n* = 7; for *iR2−/−iR1∆Ch*, P0, *n* = 5; P8, *n* = 8; P14, *n* = 8; 2m, *n* = 5). Each black dot (circle, square, triangle or diamond) in the bar graphs in panels C and D represents the data from one animal in this figure and in all subsequent figures, and the error bars represent the standard error of the mean (SEM).

Previous studies had shown that inactivation of *Adam17* in chondrocytes results in an enlarged zone of hypertrophic cells in the growth plate of long bones [15,16]. To assess the changes in the long bone growth plates of *iR2−/−iR1∆Ch* mice compared to *iR2−/−iR1fl/fl* controls at different stages of development, we performed a histological analysis of sections of the distal femur (Figure 1A,C) and proximal tibia growth plate (Supplementary Figure S1A,C) at postnatal day P0, P8, P14 and at 2 months. Analysis of sections stained with safranin O and Fast Green uncovered a significant expansion of the hypertrophic zone in the distal femoral growth plate of *iR2−/−iR1∆Ch* mice compared to *iR2−/−iR1fl/fl* controls at P0, P8, and P14 (Figure 1A, quantification in C). These differences were no longer evident at 2 months of age (Figure 1A,C). Measurements of the resting zone and the proliferating zone did not uncover significant differences between *iR2−/−iR1∆Ch* mice and *iR2−/−iR1fl/fl* controls at any of the time points analyzed, although the enlarged zone of hypertrophic chondrocytes did result in a significant increase in the total length of the growth plate at P0, P8 and P14 (Supplementary Figure S2). To determine whether the inactivation of iRhom2 affects the growth plate, we compared *iR2−/−* mice and WT littermate controls. There was no significant difference in the overall appearance of the growth plate or in the size of the hypertrophic zone in *iR2−/−* mice compared to WT controls at any of the time points examined (Figure 1B, quantification in D) or of the resting zone or proliferating zone, which was also reflected in the similar total length of the growth plate (Supplementary Figure S3). Similar results were obtained in an analysis of the tibia growth plate of *iR2−/−* mice and their WT controls (Supplementary Figure S1B,D).

### 2.2. Long Bone Growth Defects in iR2−/−iR1∆Ch Mice

**Figure 2:**
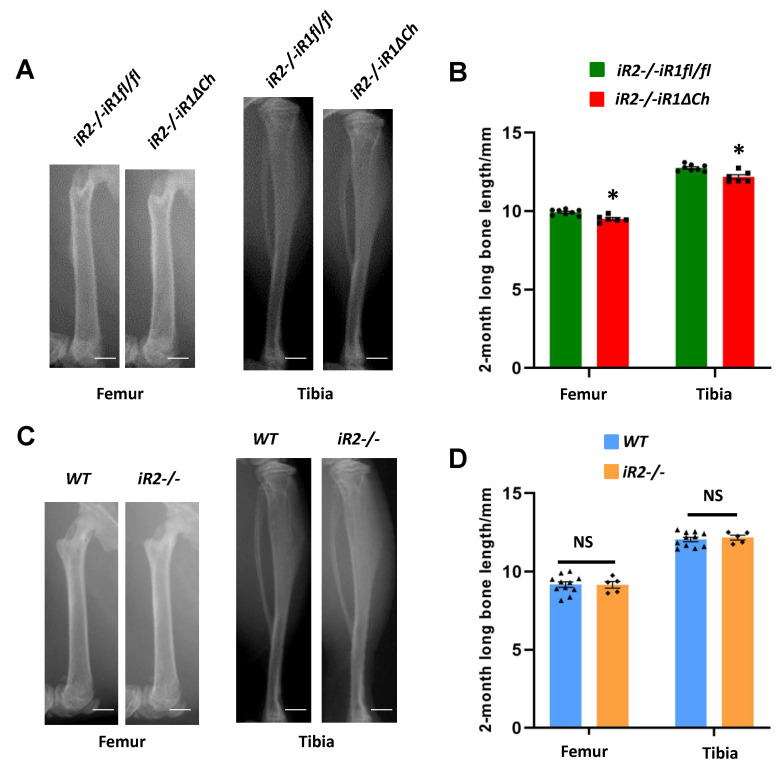
The tibiae and femurs of *iR2−/−iR1∆Ch* mice are slightly shorter than controls. (**A**) Faxitron images of a representative femur and tibia from 2-month old *iR2−/−iR1∆Ch* mice compared to the *iR2−/−iR1fl/fl* control shows that the mutant long bones are slightly, but significantly, shorter than the controls (quantification in (**B**) femurs ave. 4.3% shorter, +/− 0.86% SEM, tibiae ave. 4.5% shorter, +/− 1.1% SEM). (**C**) Faxitron images of a femur and a tibia from 2-month old WT or *iR2−/−* mice showed no significant difference in length, as quantified in (**D**). The scale bar in (**A**,**C**) is equivalent to 1 mm. * indicates a student’s *t*-test *p*-value of < 0.05, which was considered statistically significant, NS indicates no significant difference, the error bars represent the SEM. At least 5 samples from separate animals were used for quantification for each time point and genotype.

To determine whether the enlarged zone of hypertrophic chondrocytes affects bone growth in *iR2−/−iR1∆Ch* mice, we used Faxitron images to measure the lengths of the femurs and tibiae in 2-month-old mutant and control mice. Both long bones were shorter in the *iR2−/−iR1∆Ch* mice compared to their *iR2−/−iR1fl/fl* littermate controls (Figure 2A, quantification in B). In a separate analysis of the length of the femurs and tibiae of *iR2−/−* versus WT mice, we found no significant differences (Figure 2C, quantification in D).

### 2.3. Selective Expansion of Von Kossa-Stained Mineralized Hypertrophic Chondrocytes in the Growth Plate of iR2−/−iR1∆Ch Mice

**Figure 3:**
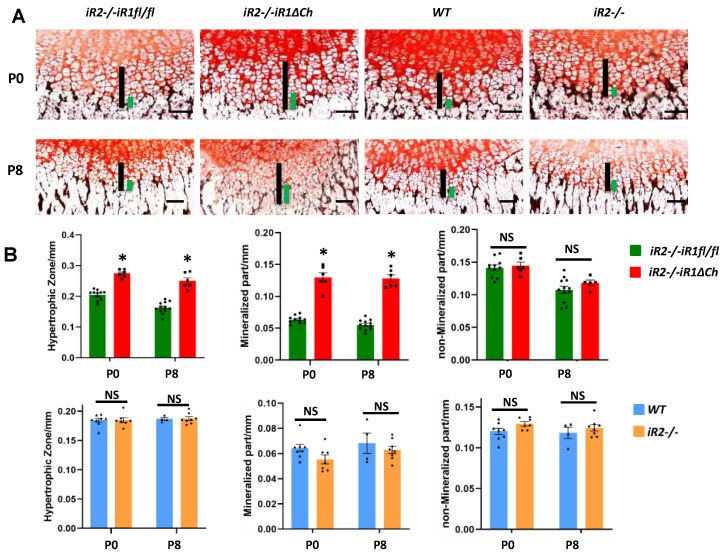
Von Kossa staining uncovers a selective enlargement of the mineralized hypertrophic chondrocytes in *iR2−/−iR1∆Ch* compared to controls. (**A**) Sections from P0 and P8 mice that were not demineralized were stained with von Kossa stain to mark the zone of mineralized hypertrophic chondrocytes adjacent to the chondro-osseous junction in black. The length of the zone of mineralized hypertrophic chondrocytes, defined as hypertrophic chondrocytes adjacent to the black von Kossa-stained material (indicated by green bars) and of the total zone of hypertrophic chondrocytes (indicated by black bars) were enlarged in *iR2−/−iR1∆Ch* mice compared to *iR2−/−iR1fl/fl* controls or compared to samples from age-matched WT or *iR2−/−* mice. (**B**) Quantification of the size of the zone hypertrophic chondrocytes and of the zone of mineralized hypertrophic chondrocytes confirmed the enlargement of both in *iR2−/−iR1∆Ch* mice compared to *iR2−/−iR1fl/fl* controls, whereas there was no significant difference in the length of the zone of non-mineralized von Kossa-negative hypertrophic chondrocytes (top panels). There was no significant difference in the length of either of these parameters between samples from age matched WT and *iR2−/−* mice (lower panels). * indicates a *p*-value of < 0.05, which was considered statistically significant, whereas NS indicates no significant difference. The scale bar indicates 100 µm. At least 4 samples from separate animals were used for each time point and genotype (WT P0, *n* = 8; P8, *n* = 4; for *iR2−/−* P0, *n* = 7; P8, *n* = 8; for *iR2−/−iR1fl/fl,* P0, *n* = 11; P8, *n* = 12; for *iR2−/−iR1∆Ch,* P0, *n* = 6; P8, *n* = 6). The error bars correspond to the SEM.

To better understand which type of chondrocyte contributes to the enlargement of the hypertrophic zone in *iR2−/−iR1∆Ch* mice, we stained sections of the distal femur growth plate from P0 and P8 mice with von Kossa stain, which stains mineralized cartilage and bone (Figure 3, black staining) and counterstained with neutral red. We then measured the length of the von Kossa-positive zone of hypertrophic chondrocytes (Figure 3A, left 2 panels, green bars) and the overall length of the zone of hypertrophic cells (Figure 3A, left 2 panels, black bars). The hypertrophic zone and the zone of von Kossa-positive mineralized chondrocytes were both significantly expanded in *iR2−/−iR1∆Ch* mice compared to *iR2−/−iR1fl/fl* controls at P0 and P8 (quantification in Figure 3B, top panels). Importantly, after subtracting the length of the mineralized von Kossa-positive component from the overall zone of hypertrophic chondrocytes, the length of the remaining non-mineralized hypertrophic zone was comparable between the *iR2−/−iR1∆Ch* mice and *iR2−/−iR1fl/fl* controls (Figure 3B, top right panel). This demonstrated that only the mineralized portion of the zone of hypertrophic chondrocytes was enlarged in *iR2−/−iR1∆Ch* mice. In a similar analysis of *iR2−/−* and WT mice, no apparent difference was found in the overall length of the hypertrophic zone and in the relative size of the mineralized zone and non-mineralized zone at P0 and P8 (Figure 3A, right two panels, Figure 3B, lower panels). These findings were confirmed in an analysis of the von Kossa-stained tibia growth plate from these animals (Supplementary Figure S4).

In principle, an expanded section of the growth plate could be the consequence of increased proliferation in the proliferating zone or decreased apoptosis at the chondro-osseus junction in the growth plate. To address these two possibilities, we performed histochemical staining of the growth plates of P0 mice with antibodies against the proliferation marker Ki-67 and with antibodies against caspase 3 as a marker for apoptosis. There were no significant differences in the percent of Ki67-positive cells in the proliferating zone of *iR2−/−iR1∆Ch* mice versus *iR2−/−iR1fl/fl* controls or of *iR2−/−* mice compared to their WT controls (quantification is shown in Supplementary Figure S5A). We also did not observe significant differences between the mutants and controls in the Ki-67 staining in the zone of hypertrophic cells, which usually does not contain proliferating cells. An analysis of caspase 3 staining also showed a comparable percentage of cells in and adjacent to the zone of hypertrophic chondrocytes that stained with this marker for apoptosis in *iR2−/−iR1∆Ch* mice compared to *iR2−/−iR1fl/fl* controls or in *iR2−/−* mice and WT controls (quantification shown in Supplementary Figure S5B). This analysis thus did not uncover evidence for a significant change in proliferation or apoptosis as a possible cause of the enlarged zone of hypertrophic chondrocytes in *iR2−/−iR1∆Ch* mice.

### 2.4. Analysis of TRAP+ Osteoclasts at the Chondro-Osseous Junction

**Figure 4:**
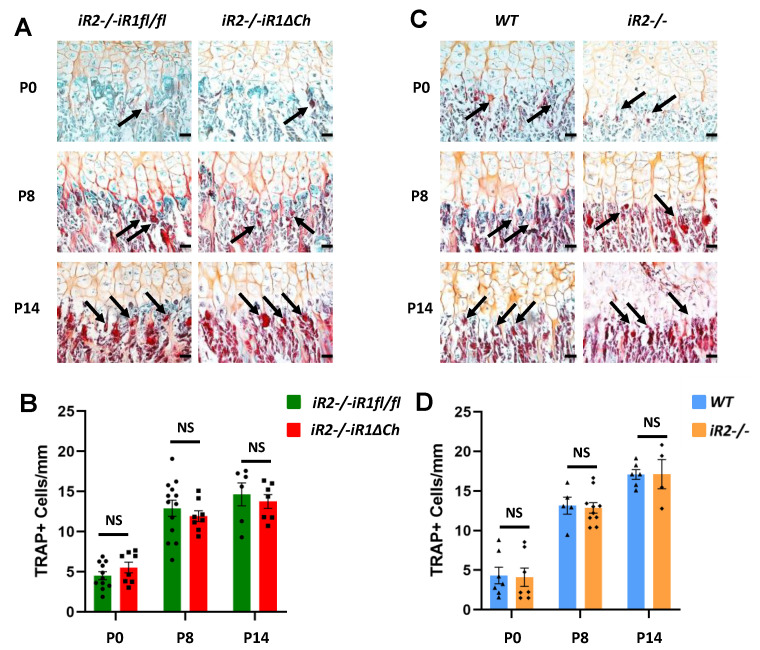
Quantification of TRAP-positive osteoclasts at the chondro-osseous junction. (**A**,**C**) TRAP stained osteoclasts (pointed by black arrows) at the chondro-osseous junction (COJ) of the distal femur growth plate of P0, P8 and P14 *iR2−/−iR1∆Ch* mice and *iR2−/−iR1fl/fl* controls (**A**) or of WT and *iR2−/−* mice (**C**) were quantified in a double blinded manner as number of TRAP+ cells per mm length of the COJ (quantification shown in (**B**,**D**)). NS indicates no significant difference, error bars represent SEM. The scale bar indicates 100 µm. A minimum of 4 samples from different animals were prepared for each genotype and time point.

Previous studies of the role of ADAM17 and of its substrate TGFα in the growth plate included an analysis of the number of tartrate-resistant acid phosphatase+ (TRAP+) cells to identify osteoclasts at the chondro-osseous junction (COJ) [15,17]. When we analyzed the number of TRAP+ osteoclasts at the COJ of *iR2−/−iR1∆Ch* mice versus controls at P0, P8 and P14, we found no significant difference at any of these time points (Figure 4A, quantification on B). Moreover, the number of TRAP+ osteoclasts at the COJ of *iR2−/−* mice versus littermate controls was also comparable (Figure 4C, quantification in D). An analysis of TRAP+ osteoclasts at the COJ of the tibiae of these animals corroborated these findings (Supplementary Figure S6). Taken together, these results suggest that the expanded zone of mineralized hypertrophic chondrocytes in *iR2−/−iR1∆Ch* mice was not caused by a reduced number of TRAP+ osteoclasts at the COJ.

### 2.5. Western Blot and qPCR Analysis of Cultured Primary Chondrocytes from iR2−/−iR1∆Ch and Control Mice

**Figure 5:**
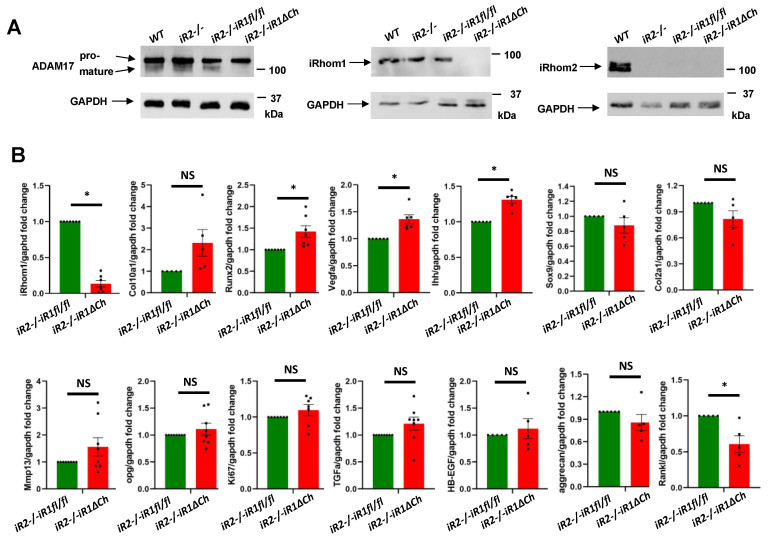
Western blot analysis of ADAM17, iRhom1 and iRhom2 expression and qPCR analysis for chondrocyte and bone markers. (**A**) Western blots of samples prepared from primary chondrocytes from WT, *iR2−/−, iR2−/−iR1fl/fl* or *iR2−/−iR1∆Ch* animals were probed for ADAM17 (left panels, see Supplementary Figure S7 for quantification), iRhom1 (middle panels) or iRhom2 (right panels). The Western blots confirmed the presence of pro-ADAM17 in all samples, whereas mature ADAM17 could only be detected in WT, *iR2−/−* and *iR2−/−iR1fl/fl* samples, but not in *iR2−/−iR1∆Ch* chondrocytes (left panels). A Western blot for iRhom1 demonstrated that it was present in primary chondrocytes from WT, *iR2−/−* and *iR2−/−iR1fl/fl* mice but had been efficiently deleted in *iR2−/−iR1∆Ch* chondrocytes (middle panels). iRhom2 could only be detected in WT primary chondrocytes, but not in chondrocytes from *iR2−/−*, *iR2−/−iR1fl/fl* or *iR2−/−iR1∆Ch* mice (right panels). In all cases, GAPDH served as a loading control. Each Western blot is representative of at least 3 separate experiments. (**B**) qPCR analysis showed an almost abolished expression of iRhom1 in *iR2−/−iR1∆Ch* chondrocytes (top left graph). There was a non-significant trend towards increased expression of Col10a1, increased expression of Runx2, Vegfa and Ihh, no significant difference in the expression of Sox9, Col2a1, Mmp13, OPG, Ki67, TGFα, HB-EGF or aggrecan, and reduced expression of Rankl in *iR2−/−iR1∆Ch* samples compared to *iR2−/−iR1fl/fl* controls. Each qPCR analysis was performed on at least 3 samples of each genotype. * indicates statistical significance with a *p*-value of <0.05 in a student’s *t*-test, whereas NS indicates no significant difference. The SEM is indicated by error bars.

To learn more about how the lack of iRhom1 and iRhom2 affects the maturation of ADAM17 in cultured chondrocytes, we isolated primary chondrocytes from the different mutant and control mice and performed Western blots for ADAM17. As shown in a representative blot in Figure 5A (left panels), pro-ADAM17 was detected in all samples, whereas mature ADAM17 was only detected in primary chondrocytes from WT, *iR2−/−* and *iR2−/−iR1fl/fl* mice, but not from *iR2−/−iR1∆Ch* mice (quantification in Supplementary Figure S7). Separate Western blots for iRhom1 and iRhom2 confirmed the expected expression or lack thereof, corresponding to the genotypes of the different primary chondrocytes (Figure 5A, middle and right panels). Thus, the Col2a-Cre-mediated inactivation of iR1 in chondrocytes appeared to be very efficient and it prevented the maturation of ADAM17, just like in *iR1/2−/−* mouse embryonic fibroblasts [30,33]. This was further corroborated by qPCR, which showed that the *iRhom1* mRNA levels were strongly reduced in *iR2−/−iR1∆Ch* chondrocytes compared to controls after 4 weeks in culture (Figure 5B). Moreover, we found a trend towards upregulation of *Col10a1*, significantly upregulated *Runx2*, *Vegf-a* and *Ihh* expression and a significant reduction in *Rankl* expression in *iR2−/−iR1∆Ch* chondrocytes versus *iR2−/−iR1fl/fl* controls. There were no significant differences in the expression of *Sox9*, *Col2a1*, *Mmp13, Opg*, *Aggrecan*, the proliferation marker *Mki-67*, or the EGFR-ligands *Tgfα* and *Hb-egf* (Figure 5B). No significant differences in the expression of the genes listed above were observed in *iR2−/−* chondrocytes versus WT chondrocytes isolated from littermate controls (Supplementary Figure S8).

### 2.6. Proteolytic Release of EGFR-Ligands from Cultured Primary Chondrocytes from iR2−/−iR1∆Ch and Control Mice

**Figure 6:**
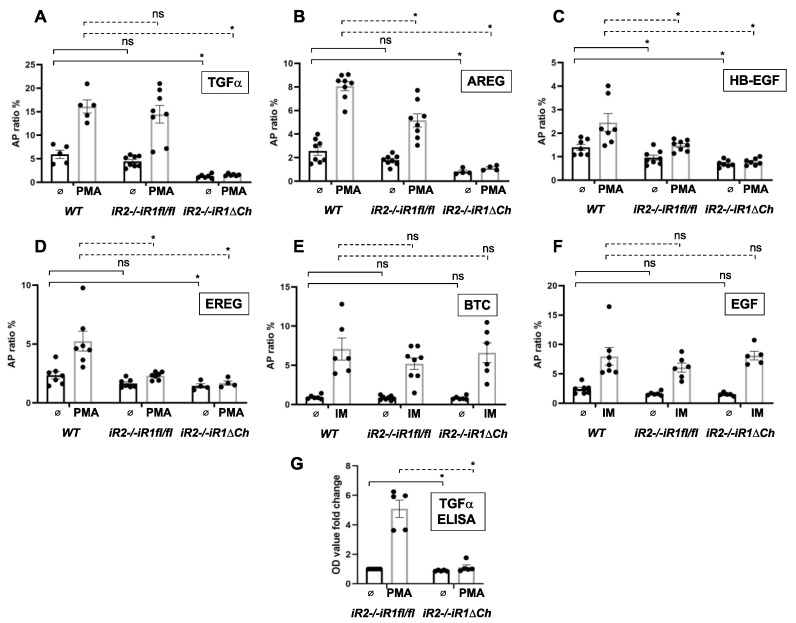
Constitutive and stimulated shedding of ligands of the EGFR from primary chondrocytes. Primary chondrocyte cultures from *WT, iR2−/−iR1fl/fl* and *iR2−/−iR1∆Ch* mice were transfected with alkaline phosphatase-tagged ligands of the EGFR (**A**–**F**) or not transfected (**G**) and then incubated for 1 h with or without 25 ng/mL of the Phorbol 12-myristate 13-acetate (PMA), which strongly stimulates shedding of the ADAM17 substrates TGFα (**A**), amphiregulin (AREG, **B**), heparin-binding EGF (HB-EGF, **C**), epiregulin (EREG, **D**), and endogenous TGFα (**G**) or with 2.5 µM of the ionophore ionomycin (IM), which stimulates both ADAM10 and ADAM17, in order to enhance the shedding of the ADAM10 substrates betacellulin (BTC, **E**) and epidermal growth factor (EGF, **F**). In all cases, the WT chondrocytes served as positive control for stimulated shedding of each EGFR-ligand. Constitutive and stimulated shedding of TGFα was not affected in *iR2−/−iR1fl/fl* compared to WT controls, but strongly reduced in *iR2−/−iR1∆Ch* chondrocytes (**A**). Stimulated shedding of AREG (**B**), HB-EGF (**C**) and EREG (**D**) was significantly reduced in *iR2−/−iR1fl/fl* compared to WT controls, and in all cases both stimulated and constitutive shedding was strongly reduced in chondrocytes from *iR2−/−iR1∆Ch* mice. Constitutive and ionomycin-stimulated shedding of the two ADAM10 substrates BTC (**E**) and EGF (**F**) was not significantly affected in either of the mutant chondrocyte cultures compared to WT controls (**E**,**F**). Finally, constitutive and PMA-stimulated shedding of endogenous TGFα was significantly reduced in *iR2−/−iR1∆Ch* chondrocytes compared to WT controls (**G**). Statistical significance, as determined by the student’s T-test, is indicated by * (*p* < 0.05), whereas NS indicates that the samples are not significantly different. The two samples compared by statistical analysis are indicted by brackets with solid lines for constitutive shedding and with dotted lines for stimulated shedding. The AP-levels in the corresponding chondrocyte lysates as a control for relative expression levels is shown in Supplementary Figure S9. The error bars indicate SEM.

ADAM17 is crucial for the shedding and functional activation of five of the seven ligands of the EGFR (TGFα, HB-EGF, AREG, EREG and EPGN) [34,35,36]. In addition, iRhom2 is responsible, at least in part, for the stimulated release of several of these EGFR ligands in mouse embryonic fibroblasts (HB-EGF, AREG and EREG) [28], whereas iRhom1 and iRhom2 can both fully support the shedding of TGFα, the main EGFR ligand responsible for normal development of the zone of hypertrophic chondrocytes [30]. When we performed assays for the activity of ADAM17 in primary chondrocytes isolated from the growth plates of WT, *iR2−/−iR1fl/fl* or *iR2−/−iR1∆Ch* mice, we found a comparable shedding of alkaline-phosphatase-tagged TGFα (TGFα-AP) from WT and *iR2−/−iR1fl/fl* cells, but not from *iR2−/−iR1∆Ch* chondrocytes. The constitutive and PMA-stimulated shedding were both strongly reduced in *iR2−/−iR1∆Ch* chondrocytes, consistent with the loss of functional ADAM17 (Figure 6A). Similar results were obtained for constitutive and PMA-stimulated shedding of AREG, which was almost completely abolished in *iR2−/−iR1∆Ch* cells. However, in *iR2−/−iR1fl/fl* chondrocytes, the PMA-stimulated shedding of AREG was reduced compared to WT controls (Figure 6B), similar to the stimulated shedding of AREG from *iR2−/−* mEFs compared to controls [28]. For HB-EGF (Figure 6C) and EREG (Figure 6D), the constitutive and stimulated shedding was strongly reduced in *iR2−/−iR1∆Ch* chondrocytes compared to WT controls and there was also a significant reduction in PMA-stimulated shedding in *iR2−/−iR1fl/fl* compared to WT chondrocytes (Figure 6C,D), consistent with previous studies in mEFs [28]. As a separate control, we examined the constitutive or ionomycin-stimulated shedding of the ADAM10 substrates BTC and EGF, which was similar in chondrocytes from all three genotypes (Figure 6E,F, please note that ADAM10 was stimulated with ionomycin since it does not respond to PMA [35,37]). Finally, when we analyzed the shedding of endogenous TGFα in primary chondrocytes by ELISA, we found strong PMA stimulation in *iR2−/−iR1fl/fl* chondrocytes, but no significant stimulation in *iR2−/−iR1∆Ch* chondrocytes (Figure 6G). These findings corroborate the results obtained with TGFα-AP overexpressed in primary chondrocytes (Figure 6A). Taken together, these functional studies demonstrate that inactivation of both iR1 and iR2 in chondrocytes abolishes the maturation and EGFR-ligand sheddase activity of ADAM17 in primary chondrocytes.

## 3. Discussion

The ADAM17/EGFR signaling pathway is known to have an important role in endochondral ossification during the growth of long bones [16,18,19,38,39]. The recent discovery that the cell surface metalloprotease ADAM17 is controlled by two essential binding partners, iRhom1 and 2 [26,27,30,31], raises interesting questions about the function of these two molecules in endochondral ossification. Previous studies have shown that simultaneous inactivation of iRhom1 and 2 in mice results in perinatal lethality and open eyes at birth as well as defects in heart valve and growth plate development, resembling the defects observed in mice lacking ADAM17 [30]. However, separately generated mice lacking iRhom1 and 2 had a more severe phenotype, resulting in early embryonic lethality, raising questions about possible functions of the iRhom2 not related to the maturation and function of ADAM17 [31]. Since the prenatal or perinatal lethality of previously described *iR1/2−/−* mice did not allow an analysis of the role of both iRhoms in postnatal endochondral ossification, we generated mice lacking iRhom2 that also carried floxed alleles of iRhom1 and the chondrocyte specific Col2a1-Cre to analyze the contribution of these two iRhoms to postnatal bone growth and endochondral ossification. Our findings that *iR2−/−iR1∆Ch* mice resemble *A17∆Ch* support the notion that iRhom1 and 2 are crucial regulators of ADAM17 during endochondral ossification.

The ability of *iR2−/−iR1∆Ch* mice to survive into adulthood without any major pathological phenotypes, like *A17∆Ch* mice, provided an opportunity to study the function of iRhom1 and 2 in endochondral ossification at different stages of postnatal mouse development. We found that *iR2−/−* mice resembled WT controls in terms of the appearance and size of the growth plate at all stages of development. Since inactivation of iRhom2 prevents the maturation of ADAM17 in myeloid cells and other immune cells [26,27,29], *iR2−/−* mice are comparable to *A17fl/fl-LysM-Cre* mice [13], which lack ADAM17 in myeloid cells and also do not have evident defects in endochondral ossification [38]. Interestingly, iRhom2 was recently implicated in the regulation of trabecular bone density, with an increase in trabecular bone described in *iR2−/−* mice [40]. However, this is presumably caused by the role of iRhom2 in osteoclasts, as these cells are derived from the myeloid lineage. This concept is further supported by the observation that mice lacking iRhom2 are protected from bone resorption in a mouse model of hemophilia arthropathy, as are mice lacking the ADAM17 substrate TNFα or mice treated with the TNF antagonist Etanercept [41]. However, our findings argue against a major role of iRhom2 in chondrocytes during endochondral ossification.

In addition to controlling the role of ADAM17 in myeloid cells, iRhom2 also affects the substrate selectivity of ADAM17 in mouse embryonic fibroblasts [28]. Similar results were obtained in this study with primary chondrocytes, in which the stimulated shedding of the EGFR ligands AREG, HB-EGF and EREG was significantly reduced in cells lacking iRhom2, whereas the stimulated shedding of TGFα, implicated in endochondral ossification [28], was not. These results suggest that the reduction or loss of iRhom2-dependent stimulated shedding of EGFR-ligands (AREG, HB-EGF, EREG) in chondrocytes does not significantly affect endochondral ossification. However, if both iRhom1 and iRhom2 were inactivated, then the shedding of TGFα from primary chondrocytes was abolished, comparable to *A17∆Ch* chondrocytes [15]. The consequences of inactivating both iRhom1 and iRhom2 in chondrocytes on the expression of marker genes (upregulation of Runx2, Vegf-a and Ihh, downregulation of Rankl) were similar to those previously described for chondrocyte lacking A17 [15], whereas no significant changes were observed in chondrocytes lacking only iRhom2. These cell biological data support the conclusion that iRhom1 and iRhom2 are crucial regulators of the role of ADAM17 in chondrocytes.

An analysis of the long bone growth plate defects in *iR2−/−iR1∆Ch* mice further confirmed the similarities to *A17∆Ch* mice. Specifically, we found an enlargement of the zone of hypertrophic cells in the growth plates of *iR2−/−iR1∆Ch* without significant changes in chondrocyte proliferation or apoptosis, very similar to *A17∆Ch* mice [15]. Moreover, von Kossa staining showed that the enlargement of the zone of hypertrophic chondrocytes was caused by a selective enlargement of the zone of mineralizing hypertrophic chondrocytes. The von Kossa-positive mineralized chondrocytes are typically only present in the final layer of hypertrophic chondrocytes adjacent to the chondro-osseous junction and do not extend more than one or two cell diameters into the cartilaginous growth plate. The finding that the von Kossa-positive zone extended further into the zone of hypertrophic chondrocytes in *iR2−/−iR1∆Ch* mice and that this expansion could account for the overall enlargement of the growth plate in these animals provided additional evidence that the growth plate defect in *iR2−/−iR1∆Ch* mice resembles that in *A17∆Ch* mice [15]. This was corroborated by a Western blot analysis, which showed a lack of iRhom1 and of mature ADAM17 in chondrocytes from *iR2−/−iR1∆Ch* mice compared to chondrocytes from *iR2−/−* or *iR2−/−iR1fl/fl* mice or WT controls. These findings provide a plausible explanation for why long bones (femur and tibia) were significantly shorter in *iR2−/−iR1∆Ch* mice compared to controls, just like in *A17∆Ch* animals. Since the main phenotype of *iR2−/−iR1∆Ch* mice is an enlarged area of mineralizing hypertrophic chondrocytes without evident changes in the adjacent bone at the chondro-osseus junction, we feel that the phenotype is likely not related to an effect on the ability of hypertrophic chondrocytes to transdifferentiate into osteoblasts [42,43,44].

Taken together, this study uncovered an essential role for iRhom1 and iRhom2 in the maturation of ADAM17 in chondrocytes and in the regulation of endochondral ossification during postnatal long bone growth. These results lend further support to the interpretation that both iRhoms are crucial regulators of ADAM17, with redundant or compensatory functions, since mice lacking only iRhom1 or iRhom2 develop normally without any major spontaneous defects in endochondral ossification or otherwise [26,27,30]. ADAM17, in turn, is thought to regulate endochondral ossification by releasing the EGFR-ligand TGFα, which can be processed by both iRhom1/ADAM17 and iRhom2/ADAM17 [28], but is no longer released by primary chondrocytes lacking both iRhom1 and iRhom2. These results thus provide novel insights into the molecular components and mechanisms underlying endochondral bone formation. The translational relevance of these findings is that potential defects in the interaction of ADAM17 with both iRhoms could conceivably be a cause of abnormal bone growth in humans. Moreover, since both iRhoms are involved in the maturation and posttranslational activation of ADAM17, further studies of how the iRhoms regulate ADAM17 could uncover new approaches to modulate endochondral ossification and perhaps bone healing.

## 4. Materials and Methods

### 4.1. Reagents and Antibodies

All materials were from Sigma-Aldrich (St. Louis, MO, USA) unless noted otherwise. ADAM17 was detected with rabbit polyclonal antibodies against its cytoplasmic domain [45]; iRhom1 was detected with anti-iRhom1/RHBDF1 #20A8 rat IgG2a [46]; iRhom2 was detected with anti-iRhom2/RHBDF2 #11h7 [46]. HRP-labeled anti-rabbit IgG and anti-mouse IgG were from Promega, (Madison, WI, USA, # W401B (anti-rabbit) and # W402B (anti-mouse)), the anti-GAPDH antibody was from ABclonal (Woburn, MA, USA, catalog # AC002) and HRP-conjugated rabbit anti-rat antibodies were from Sigma-Aldrich, St Louis, MO, USA (# A5795). dNTPs for qPCR and genotyping were from Qiagen (Hilden, Germany).

### 4.2. Mouse Strains

*iRhom2−/−* and WT control littermate mice were generated from *iRhom2+/*− parents on a 129Sv/C57BL/6 mixed background [27]. *iRhom2−/−iRhom1fl/fl/Col2a1-cre* mice (referred to as *iR2−/−iR1∆Ch* mice) were generated by crossing *iRhom2−/−* mice with *iRhom1fl/fl* mice [30] as well as with *Col2a1-Cre* mice [32] (kindly provided by Dr. Hicham Drissi, University of Connecticut, Farmington, CT, USA) with *iR2−/−iR1fl/fl* mice serving as controls. Genotyping for *iRhom1fl/fl* and *iRhom2−/−* was performed as described previously [27,30]. Genotyping for *Col2a1-Cre* (B6;SJL-Tg(Col2a1-cre)1Bhr/J) was performed as described by Jackson Laboratories. Matings were set up such that the *Col2a1-Cre* transgene was only carried on one allele in the males, and all comparisons were performed between littermates, with an equal distribution of males and females. All animal experiments were approved by the Internal Animal Care and Use Committee of Weill Cornell Medicine (Project identification number: 2015-0047, Approval date: 4 December 2019).

### 4.3. Sample Processing

Knees were isolated at postnatal day 0 (P0), P8, P14 and 2 months from *iRhom2−/−*, *iR2−/−iR1∆Ch* mice and their respective controls, fixed overnight at 4 °C with 4% paraformaldehyde (PFA), prepared from a 16% Formaldehyde Solution (Thermo-Fisher, Waltham, MA, USA) that was diluted 1:4 in PBS and then washed 3× with PBS. P14 and 2-month old samples were decalcified with 20% EDTA (pH 7.4) for 7 days at room temperature, then embedded in paraffin and 7 µm sections were floated in a 40 °C water bath and captured on Superfrost Plus Microscope Slides (Cardinal Health, Dublin, OH, USA). The slides were dried in a 37 °C degree incubator overnight before staining.

### 4.4. Safranin O and Fast Green Staining

Safranin O and Fast Green staining for cartilage was performed on the paraffin-embedded sections following standard protocols [15]. Safranin O stains growth plate cartilage red and Fast Green serves as a counterstain to provide a better contrast. The images were generated using a Nikon Labophot microscope (Tokyo, Japan) and imported into NIH ImageJ (ImageJ bundled with 64-bit Java 1.8.0_172, National Institutes of Health, Bethesda, MD, USA) to measure the length of the different zones of chondrocytes in the growth plate, using an image of a ruler on a microscope slide as a reference for normalization. The lengths of the growth plate zones (zone of resting, proliferating, and hypertrophic chondrocytes) were measured by a double blinded observer and were distinguished by the unique morphology of the chondrocytes residing in different zones (resting zone—small and round; proliferating zone—stacked and disc-like; hypertrophic zone—significantly enlarged) [39].

### 4.5. Von Kossa Staining

Von Kossa staining was performed on knee joints of P0 and P8 animals. The sections were deparaffinized, dehydrated, rinsed with distilled water, then incubated with 1% Silver Nitrate solution and exposed to UV light for 15 min in a Bio-Rad UV transilluminator (Hercules, CA, USA). The unreacted silver was removed by treatment with 5% sodium thiosulfate for 5 min, followed by counterstaining with 1% neutral red for 1 min.

### 4.6. TRAP Staining

Tartrate-resistant acid phosphatase (TRAP) staining was performed on paraffin sections of knees from P0, P8, P14 and 2-month-old mice. The sections were incubated in Basic Stock Incubation Solution (BSIS, Sodium acetate, Sodium tartrate and Acetic acid, pH 4.7–5.0) with Napthol AS-BI phosphate dissolved in 2-Ethoxyethanol at 37 °C degree for 1 h. The samples were then rinsed in BSIS with Sodium Nitrite and Pararosaniline dye and developed at 37 °C until the desired intensity of the red staining in osteoclasts was achieved. The samples were counterstained with 0.02% Fast Green for 2.5 min. TRAP+ cells at the COJ were counted using a Nikon Labophot microscope (Tokyo, Japan) at 200× magnification on at least 2 sections per animal.

### 4.7. Immunohistochemistry

Slides with paraffin sections of growth plates of P0 mice were subjected to immunohistochemistry for the proliferation marker Ki67 (used at 1:25 dilution, mouse monoclonal, clone BGX-297, Biogenex, San Ramon, CA, USA) and for the apoptosis marker cleaved caspase-3 (Cell Signaling Technology; catalog # 9661, used at a 1:250 dilution in heat-induced epitope retrieval buffer (HIER), pH 6.0,) according to the manufacturer’s protocols. For quantification of chondrocyte proliferation using Ki67 immunohistochemical (IHC) staining, a rectangular segment (800 µm × 200 µm) in the center of the proliferating zone of chondrocytes of the growth plate was chosen to count the total number of cells, using the cell count function in NIH ImageJ and then the number of cells with brown nuclei (Ki67 positive). The percentage of proliferating cells was calculated by the dividing the number of Ki67 positive cells by the total number of cells and multiplying by 100. For cleaved caspase-3 IHC staining, all cells and all positively stained nuclei in an area ranging from 100 µm above to 100 µm below the COJ (for a total width of 200 µm) along the length of the COJ were counted separately using the cell count function in NIH ImageJ. The number of nuclei was divided by the length of the COJ in µm and multiplied by 100 to calculate the number of cleaved caspase-3 positive cells per 100 µm of COJ. All slides were photographed using a Nikon Labophot microscope (Tokyo, Japan) and at least 2 sections were captured per animal.

### 4.8. Faxitron Analysis

Radiographic images of limbs were generated for 2-month adult mice using a Faxitron (Tucson, AZ, USA, Specimen DR software version 3.1.0) and were analyzed with NIH ImageJ software to measure the longest distances from the proximal to the distal joint of the tibia and femur.

### 4.9. Cell Culture and Protein Ectodomain Shedding Assays

Primary chondrocytes were isolated from the femur head, distal femur and proximal tibia from 4 to 6 days old mice as described previously [47]. Passage 0 to 2 chondrocytes were used for experimental purposes. The cells were maintained in high density monolayer cultures for 4 weeks in complete medium containing ascorbic acid, which has been shown to lead to increased expression of hypertrophic markers in monolayer and pellet cultures [15,48,49]. Briefly, cells were plated at a density of 2.5 × 10^4^ cells/cm^2^ in Dulbecco’s Modified Eagle Medium (DMEM) F-12 with 10% fetal bovine serum (Atlanta Biologicals, Flowery Branch, GA, USA), 1% penicillin/streptomycin, 1× ITS universal culture supplement (containing insulin, transferrin and selenous acid; BD Biosciences, Franklin Lakes, NJ, USA), and 50 ug/mL of ascorbic, with changes of medium every 48 h. After 4 weeks, chondrocyte proliferation was assessed by real-time quantitative reverse transcription-PCR (qRT-PCR) analysis for *Mki67*, encoding the Ki67 antigen and for *Runx2* and *Mmp13* mRNA to assess hypertrophic differentiation. Protein ectodomain shedding experiments with transfected alkaline phosphatase-tagged EGFR ligands were performed as described previously [35,50], except that the primary chondrocytes were transfected with Lipofectamine 3000 (Thermo-Fisher, Waltham, MA, USA). Briefly, 1 × 10^5^ freshly isolated primary chondrocytes from WT, *iR2−/−, iR2−/−iR1fl/fl* or *iR2−/−iR1∆Ch* mice were plated out per well of 12 well tissue culture plates and cultured overnight in DMEM F12 (see above, but without Ascorbic acid and ITS). The next day, plasmids encoding alkaline-phosphatase (AP) tagged TGFα, HB-EGF, AREG, EREG, EGF and BTC [35,50] were separately transfected into primary chondrocytes with Lipofectamine 3000 for 5 h in reduced serum OPTI-MEM (OPTI-Eagle’s minimal essential medium, Thermo-Fisher, Waltham, MA) and then the cells were cultured in DMEM F-12 overnight. For the shedding experiment on the next day, the cells were serum starved in OPTI-MEM for 30 min, then fresh OPTI-MEM was added with or without 25 ng/mL PMA (Phorbol 12-myristate 13-acetate) for one hour to stimulate iRhom/ADAM17-dependent ectodomain shedding of the ADAM17-dependent AP-tagged EGFR-ligands (TGFα, HB-EGF, AREG, EREG [36,50]) by proteolytic processing at their juxtamembrane cleavage site. Since the activity of ADAM10 is not activated by PMA, shedding of the two ADAM10 substrates EGF and BTC was stimulated by addition of 2.5 µM ionomycin, which strongly stimulates ADAM10 [37,51]. The alkaline phosphatase substrate, 4-nitrophenyl phosphate, was added to the supernatant and cell lysate and the absorbance at A405 nm was measured to assess the AP activity [36]. Measurements were conducted in triplicates and the ratio between the AP activity in the supernatant and the AP-activity in the cell lysate plus supernatant was calculated. Each experiment was performed at least three times per genotype with a total of three wells per experiment.

### 4.10. Western Blot Analysis

For Western blotting to determine the levels of pro- and mature ADAM17 or of iRhom1 or iRhom2 in primary chondrocytes after 4 weeks in culture, the cells were lysed in 1% Triton X-100, 50 nM Tris HCl pH 7.4, 150 mM NaCl, 1 mM EDTA, 10 mM NaF, 2 mM NaVO3, 1 mM 1,10-phenanthroline (the latter was added to prevent post-lysis autodegradation of mature ADAM17 [27,45]), and 1× protease inhibitor cocktail (Roche, Basel, Switzerland) in PBS. The cell lysate was cleared by centrifugation at 15,000× *g* for 5 min and glycoproteins were concentrated on Concanavalin A-Sepharose beads (GE Healthcare, Chicago, IL, USA) for Western blots of ADAM17 (25 µL of a 50% Con-A bead slurry per 1 mL of lysate). The glycoproteins bound to Concanavalin A-Sepharose beads were removed by boiling in SDS-sample loading buffer for 5 min, then reduced with beta-mercaptoethanol (1:10). For Western blots of iRhom1 and iRhom2, the cell lysates were prepared as described above, but without enrichment of glycoproteins on Concanavalin A-Sepharose beads. All samples were separated on 10% SDS-PAGE gels and transferred to nitrocellulose membranes. ADAM17 was detected with an antibody against its cytoplasmic domain [45], whereas iRhom1 and iRhom2 were detected with rat monoclonal antibodies that have been described previously [46]. NIH Image J was used for quantification of the relative level of pro- or mature ADAM17 in Western blots after subtraction of non-specific background levels.

### 4.11. TGFα ELISA

To measure endogenous TGFα released from confluent primary chondrocytes (genotypes: *iR2−/−iR1∆Ch* or *iR2−/−iR1fl/fl* controls), the cells were cultured in a 10-cm tissue culture plate for 24 h and then washed and starved in 5 mL OPTI-MEM for one hour, at which point they were either treated with 25 ng/mL PMA or left untreated for an additional hour. TGFα released into the culture supernatant was measured with an ELISA kit for human TGFα (Duoset ELISA DY239, R&D Systems, Minneapolis, MN, USA), which also detects mouse TGFα [52], following to the manufacturer’s instructions. TGFα in the supernatants was concentrated by ethanol precipitation. Briefly, 5 mL supernatant was harvested from the plates, then 45 mL of 100% Ethanol (−20 °C) was added, mixed and kept at −80 °C overnight. Then, the samples were spun at 15,000× *g* in an SS-34 rotor in a Sorvall Evolution centrifuge for 15 min at 4 °C. The supernatant was aspirated and the pellet washed in 90% Ethanol (−20 °C), vortexed and spun again for 5 min at 15,000× *g*. Then, the supernatant was removed and the pellets were dried at 55 °C for 15 min and then resuspended in 400 µL PBS + 0.05% Tween 20. A quantity of 100 µL of the resuspended solution was used to measure the concentration of endogenous mouse TGFα in triplicate samples in an ELISA plate.

### 4.12. qRT-PCR Analysis

For qRT-PCR analysis, total RNA was isolated from primary chondrocytes cultured in monolaters for 4 weeks after reaching confluence. Total RNA was isolated using TRIzol Reagent (Life Technologies, Grand Island, NY, USA) and chloroform, after spinning the samples for 15 min at 12,000× *g* at 4 °C. The RNA was isolated from the supernatant using isopropanol, then washed in 75% ethanol and dissolved in purified water (Millipore Molecular Biology Grade Water, MilliporeSigma, Burlington, MA, USA). Total RNA was reverse transcribed into cDNA using 2.5 mM dNTPs, RNasin Plus (Promega, Madison, WI, USA) and M-MuLV Reverse Transcriptase and Random Primer-9 (New England Biolabs, Ipswich, MA, USA). Gene amplifications were carried out using SYBR Green I-based real-time PCR, using primers for Mki-67 from Qiagen (Hilden, Germany), catalog number QT00247667, and the specific primers indicated in Table 1 [15]. The data were calculated as the ratio of each gene to *Gapdh* using the 2^−ΔΔCt^ method for relative quantification.

### 4.13. Statistical Analysis

Quantification of histopathological samples and faxitron images was performed in a double blinded manner. All graphs are presented as mean ± SEM with data from at least three independent experiments, analyzed with Prism GraphPad 8.4.3. For each animal, four representative sections of the growth plates of the left proximal tibia and of the left distal femur and four representative sections of the growth plates of the corresponding right proximal tibia and right distal femur were analyzed. Each growth plate section was measured in 3 positions (at the center and on either side halfway between the center and the edge of the growth plate). Thus, a total of 8 growth plate sections were measured for each bone type (4 left + 4 right proximal tibia sections and 4 left + 4 right distal femur sections) per animal and time point. Statistical significance was calculated using the unpaired Student’s *t* test, with a *p* value of < 0.05 considered significant (indicated by *). The statistical analysis of the qRT-PCR results was performed by the Wilcoxon–Mann–Whitney test for paired samples, with *p* < 0.05 considered statistically significant (indicated by *).

## Figures and Tables

**Table 1:** Primers for qPCR of mouse genes.

Gene	Primers Sequence for qPCR Amplification	Direction	NT
*iRhom1*	5′-TCC ACG TAC CCA GAT GAG GTG-3′	Forward	21
	5′-GCC TTC CTT CTG CTT TCT CCA-3′	Reverse	21
*Col10a1*	5′-ACG CAT CTC CCA GCA CCA GAA TC-3′	Forward	23
	5′-GGG GCT AGC AAG TGG GCC CT-3′	Reverse	20
*Runx2*	5′-TCC CCG GGA ACC AAG AAG GCA-3′	Forward	21
	5′-AGG GAG GGC CGT GGG TTC TG-3′	Reverse	20
*Vegfα*	5′-CTC GCA GTC CGA GCC GGA GA-3′	Forward	20
	5′-GCA GCC TGG GAC CAC TTG GC-3′	Reverse	20
*Ihh*	5′-GTC AAG TCT GAG CAT TCG GC-3′	Forward	20
	5′-CAT CAC TGA AGG TGG GGG TC-3′	Reverse	20
*Mmp13*	5′-ATG GTC CAG GCG ATG AAG ACC CC-3′	Forward	23
	5′-GTG CAG GCG CCA GAA GAA TCT GT-3′	Reverse	23
*Tnfrsf11b*	5′-ACA GTT TGC CTG GGA CCA AA-3′	Forward	20
*(OPG)*	5′-TCA CAG AGG TCA ATG TCT TGG A-3′	Reverse	22
*Rankl*	5′-CGA GCG CAG ATG GAT CCT AA-3′	Forward	20
	5′-CCC CCT GAA AGG CTT GTT TC-3′	Reverse	20
*TGFα*	5′-CTC TGG GTA CGT GGG TGT TC-3	Forward	20
	5′-CTG ACA GCA GTG GAT CAG CA-3′	Reverse	20
*Hb-egf*	5′-CCT CTT GCA AAT GCC TCC CT-3′	Forward	20
	5′-ACA AGA AGA CAG ACG GAC GAC-3′	Reverse	21
*Gapdh*	5′-GGG CTC ATG ACC ACA GTC CAT GC-3′	Forward	23
	5′-CCT TGC CCA CAG CCT TGG CA-3′	Reverse	20
*Sox9*	5′-AAG CTC TGG AGG CTG CTG AAC GAG-3′	Forward	24
	5′-CGG CCT CCG CTT GTC CGT TCT-3′	Reverse	21
*Col2a1*	5′-CGA TCA CAG AAG ACC TCC CG-3′	Forward	20
	5′-GCG GTT GCA AAG TGT TTG GC-3′	Reverse	20
*Acan*	5′-GGT CAC TGT TAC CGC CAC TT-3′	Forward	20
	5′-CCC CTT CGA TAG TCC TGT CA-3′	Reverse	20

## Supplementary Materials

The following are available online at https://www.mdpi.com/1422-0067/21/22/8732/s1.
