# Supplementary material for: Role of iRhoms 1 and 2 in Endochondral Ossification
Source: Int J Mol Sci. 2020 Nov 19;21(22):8732. doi: 10.3390/ijms21228732 (PMC7699240; doi:10.3390/ijms21228732)
Supplement: Supplementary file 1 [file ijms-21-08732-s001.pdf]

International Journal of
Molecular Sciences

Supplementary Figures for the article entitled

Role of iRhoms 1 and 2 in endochondral ossification

by Renpeng Fang et al.

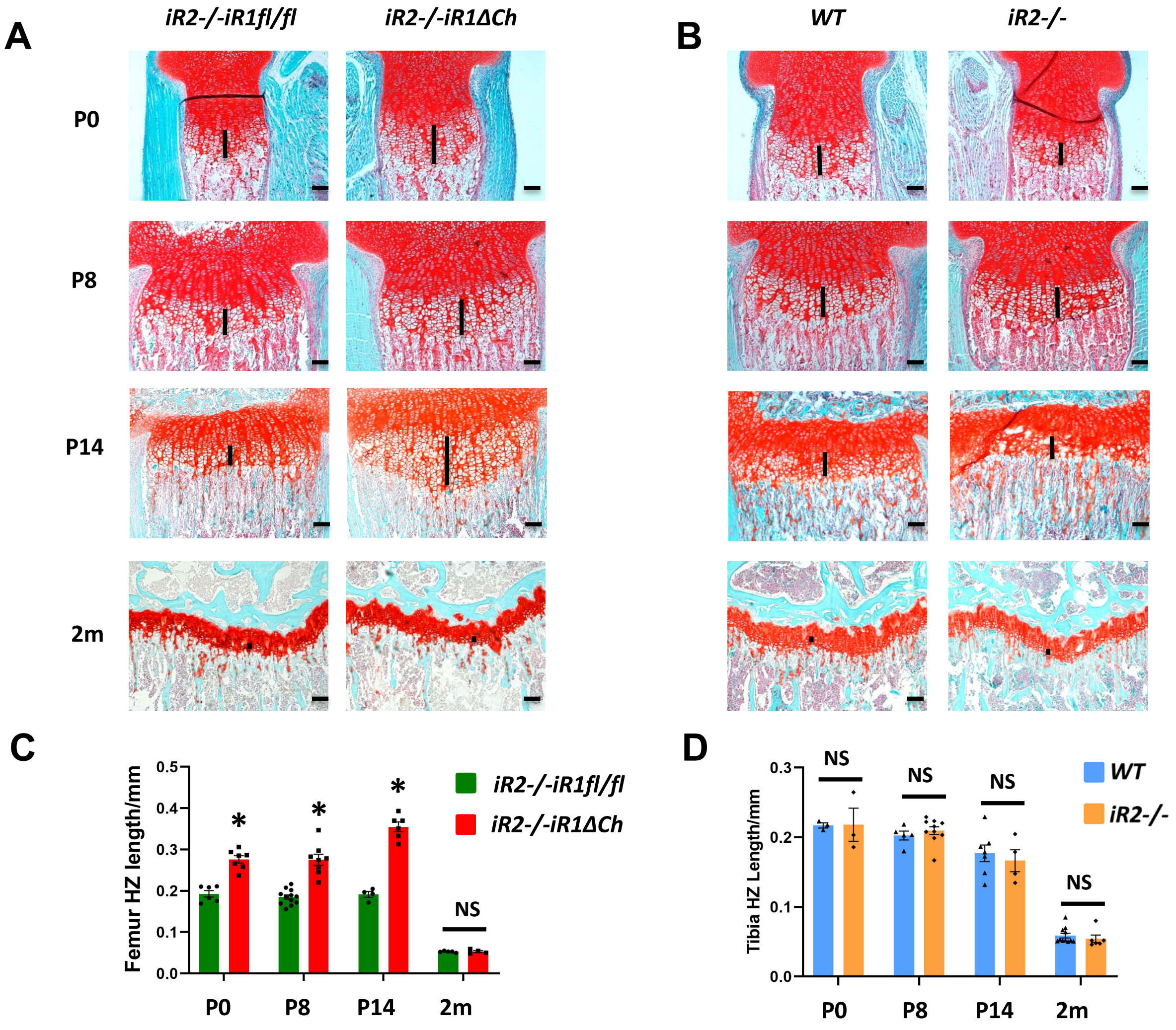

Supplementary Figure 1. Enlarged zone of hypertrophic chondrocytes in the proximal tibial growth plate of *iR2-/-iR1ΔCh* mice compared to controls. A, B) Safranin O and Fast Green-stained sections of the proximal tibial growth plate from *iR2-/-iR1fl/fl* controls or *iR2-/-iR1ΔCh* mutants (A) or from wild type (WT) or *iR2-/-* mice (B) at P0, P8, P14 and 2 months (m). A black line marks the zone of hypertrophic cells. C, D) Quantification of the increased length of the zone of hypertrophic chondrocytes at P0, P8, P14 and 2 months in *iR2-/-iR1ΔCh* samples and *iR2-/-iR1fl/fl* controls (C) and in WT and *iR2-/-* samples (D). Scale bars: 100 μm. *: Student's t-test P-value of <0.05, NS: no significant change. Quantification was on samples from 3 or more separate animals per time point and genotype, error bars: SEM.

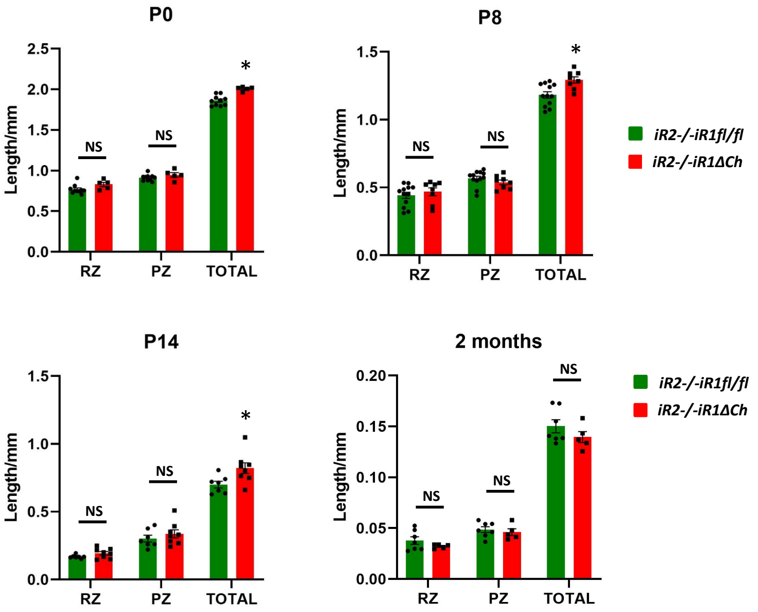

Supplementary Figure 2. Measurement of the length of different zones of chondrocytes in the growth plate of *iR2-/-iR1ΔCh* mice compared to controls at different ages. The length of the resting zone (RZ) and proliferating zone (PZ) in samples from *iR2-/-iR1ΔCh* mice and age matched *iR2-/-iR1fl/fl* mice was comparable at different ages (P0, P8, P14, 2 months), whereas the overall length of the femur growth plates was significantly larger than controls, suggesting that the significantly increased overall length of the growth plate was caused by an increased length of the zone of hypertrophic chondrocytes (see Figure 1), but not by changes in the zone of resting or of proliferating chondrocytes. An asterisk indicates a student's t-test P-value of <0.05, NS: no significant change. Samples from at least 4 animals were quantified per time point, error bars: SEM.

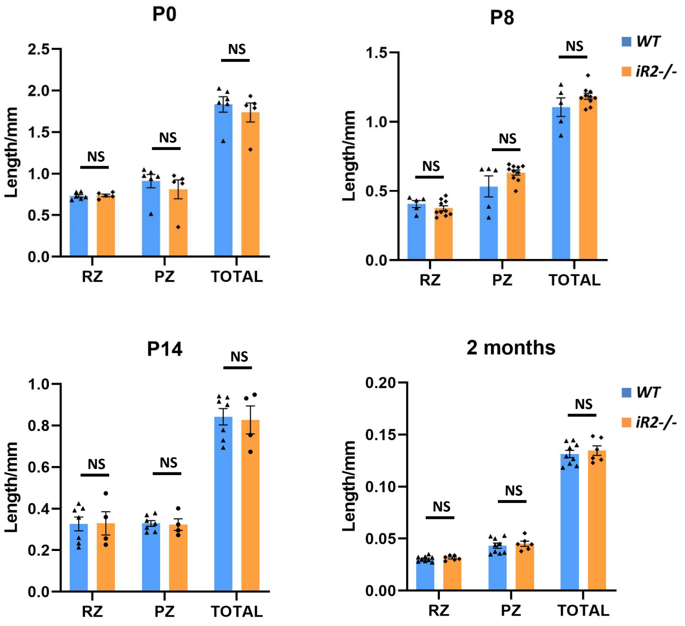

Supplementary Figure 3. Determination of the length of different zones of chondrocytes in the growth plate of *iR2-/-* mice compared to WT controls at different ages. Measurements of the length of the resting zone (RZ), the proliferating zone (PZ) and the total length of the chondrocyte-containing zone in growth plates from *iR2-/-* mice and age matched *WT* controls showed no significant (ns) difference at any of the time points

examined (P0, P8, P14, 2 months), as determined by student's T-test on at least 5 samples from different animals per genotype (significance: p<0.05). The error bars indicate SEM.

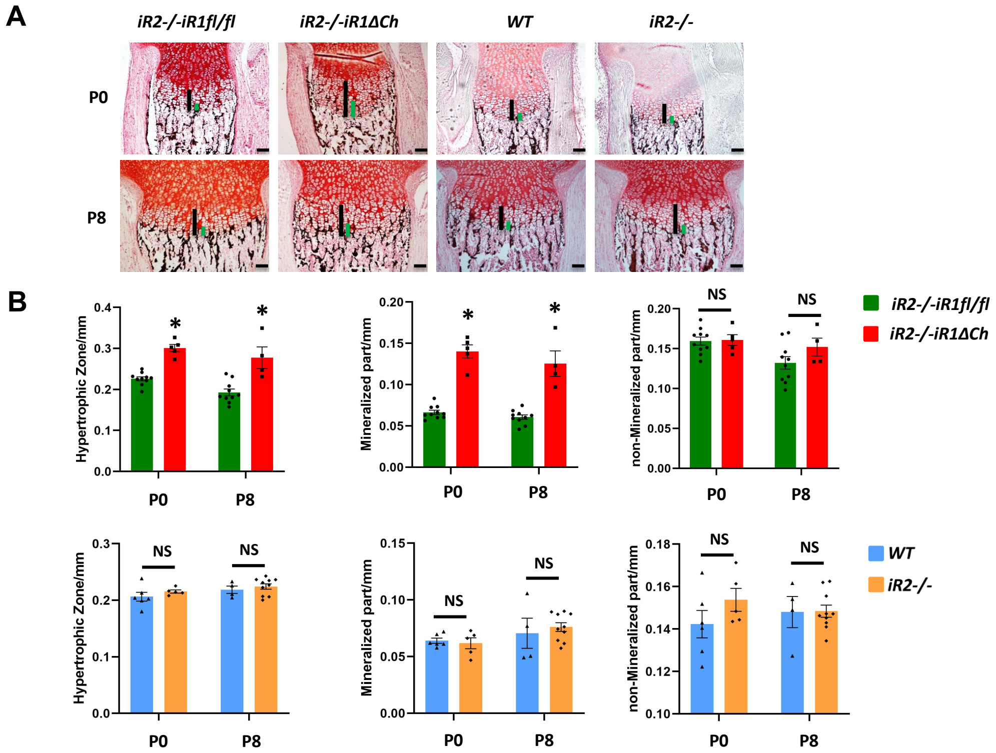

Supplementary Figure 4. The zone containing von Kossa-positive mineralized hypertrophic chondrocytes is selectively enlarged in the proximal tibial growth plate in *iR2-/-iR1ΔCh* compared to controls. A) Sections of the proximal tibial growth plate from P0 and P8 animals were stained with von Kossa to identify the zone of mineralized hypertrophic chondrocytes by the black staining. The length of the zone of hypertrophic von Kossa-positive chondrocytes (marked in green) and the total zone of hypertrophic chondrocytes (marked in black) was increased in *iR2-/-iR1ΔCh* mice compared to *iR2-/-iR1fl/fl* controls or to age matched WT or *iR2-/-* mice. B) Quantification of results obtained with *iR2-/-iR1ΔCh* mice compared to *iR2-/-iR1fl/fl* controls (top panel), and of samples from age matched WT and *iR2-/-* samples (lower panels). * P-value of <0.05 in a student's t-test, NS, no significant difference, scale bar 100 μm. At least 4 samples from separate animals were used per genotype and time point, error bars: SEM.

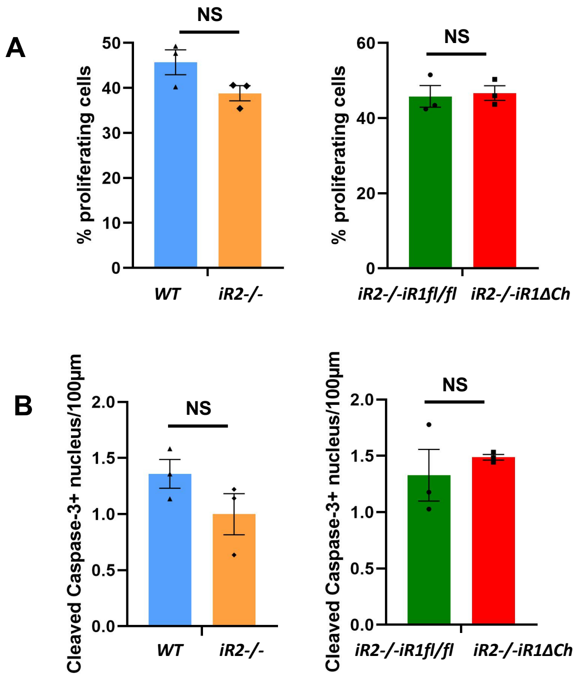

Supplementary Figure 5. Evaluation of chondrocyte proliferation using Ki67 staining and of Apoptosis using cleaved caspase 3 staining of the growth plate. A) The percentage of proliferating Ki67-positive chondrocytes in the growth plate was comparable in *WT* and *iR2-/-* mice as well as in *iR2-/-iR1fl/fl* and *iR2-/-iR1ΔCh* mice (see materials and methods for details). B) The quantification of the number of cells along the chondro-osseus junction that were positive for the apoptosis marker cleaved caspase 3 showed no significant difference in the growth plate of *WT* versus *iR2-/-* mice or in *iR2-/-iR1fl/fl* versus *iR2-/-iR1ΔCh* samples. NS indicates no significant different between the indicated samples, as determined by student's t-test, with $p<0.05$ considered statistically significant. The error bars indicate SEM.

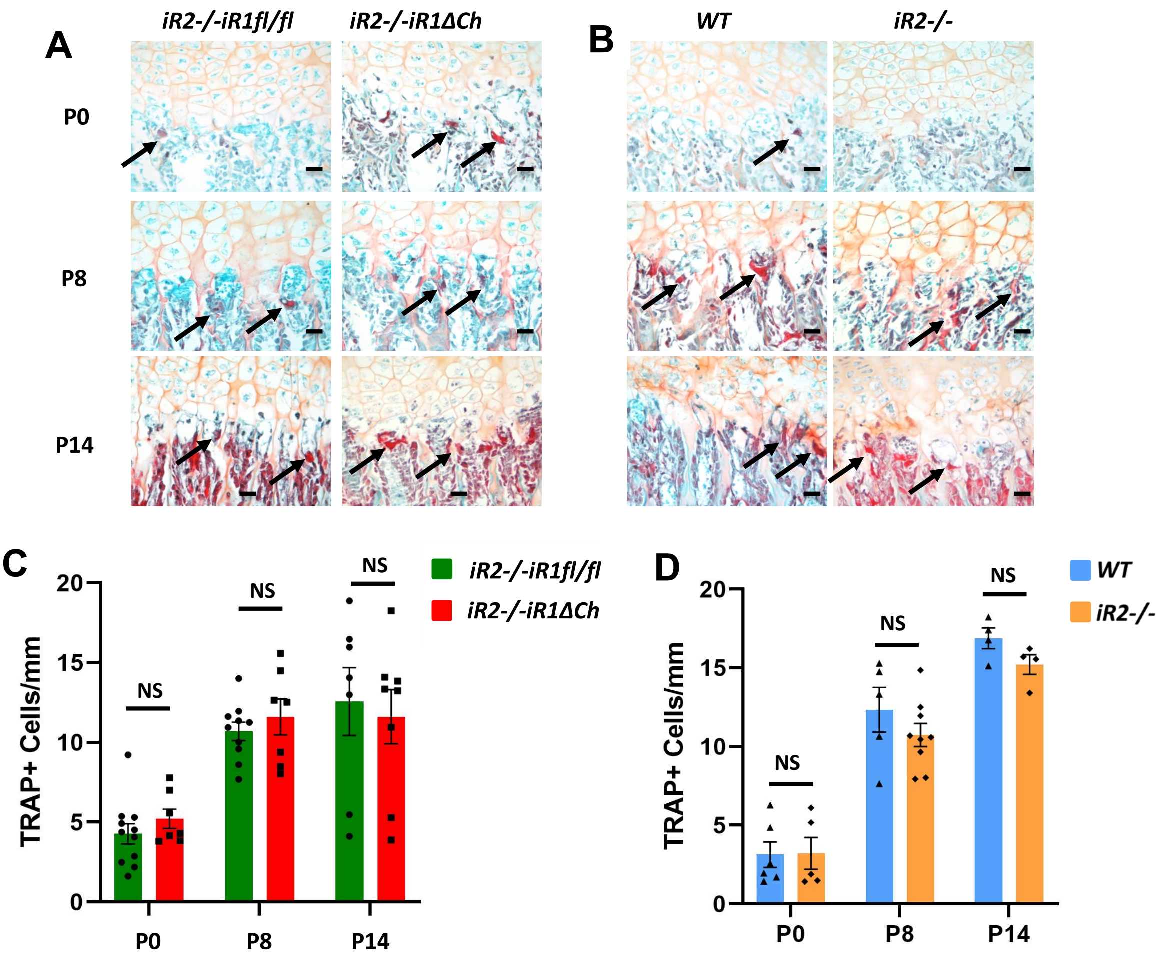

Supplementary Figure 6. Staining for TRAP-positive osteoclasts at the chondro-osseous junction of the proximal tibial growth plate. A, B) Osteoclasts marked by TRAP staining (black arrows) at the COJ of the proximal tibia growth plate at P0, P8 and P14 *iR2-/-iR1ΔCh* mice and *iR2-/-iR1fl/fl* controls (A) or WT and *iR2-/-* mice (B). C, D) Quantification of TRAP+ cells at the COJ (cells per mm). NS, no significant difference, error bars: SEM, scale bars: 100 μm. At least 4 samples were analyzed for each genotype and time point.

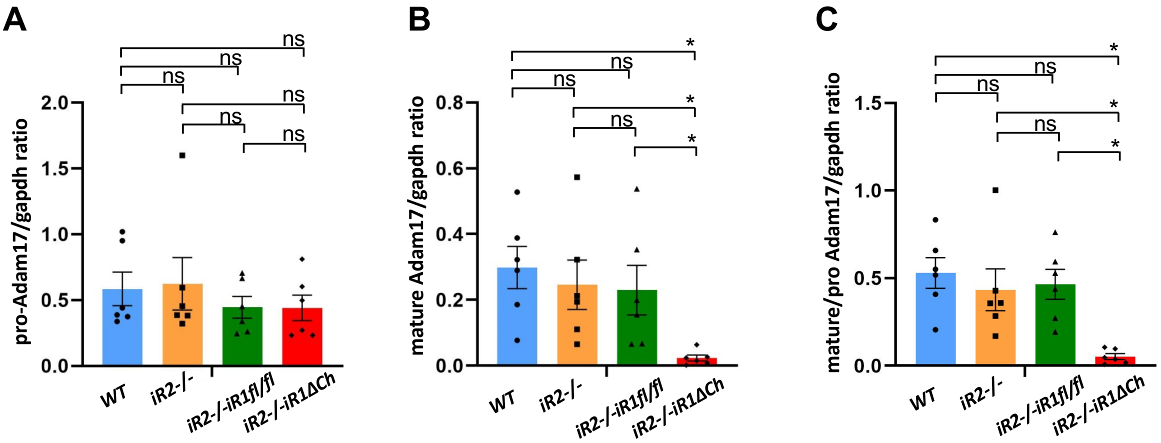

Supplementary Figure 7. Densitometric quantification of the levels of pro- and mature ADAM17 in Western blots of ADAM17 in samples from chondrocytes with the different genotypes used in this study. Densitometric measurements of pro- and mature ADAM17 as well as the GAPDH loading control were performed on six Western blots for ADAM17 in lysates of primary chondrocytes isolated from three separate litters of WT, *iR2-/-*, *iR2-/-iR1fl/fl* and *iR2-/-iR1ΔCh* mice (please see Materials and Methods for culture conditions). The resulting values were used to calculate the ratio between pro-ADAM17 and GAPDH (A),

mature ADAM17 and GAPDH (B) and mature ADAM17 and pro-ADAM17 (C). A representative Western blot is shown in the left panel of Figure 5A. The error bars correspond to the SEM. ns, no significant difference, * indicates P <0.05 in a student's t-test.

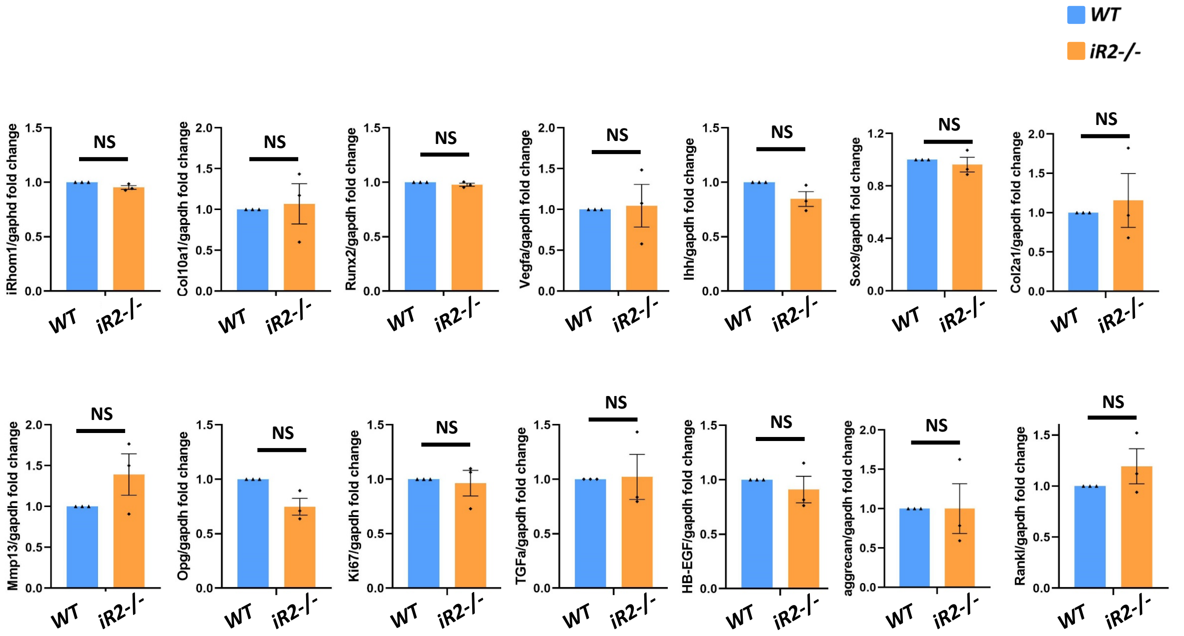

Supplementary Figure 8. qPCR analysis for chondrocyte and bone markers in *WT* versus *iR2-/-* chondrocyte cultures. qPCR analysis showed no significant difference in the expression of iRhom1, Col10a1, Runx2, Vegfa, Ihh, Sox9, Col2a1, Mmp13, OPG, Ki67, TGFα, HB-EGF, aggrecan and RANKL in *iR2-/-* chondrocytes versus *WT* controls. The qPCR analysis was performed on at least 3 samples per genotype and gene analyzed. NS indicates no significant statistical difference (P >0.05 in a student's t-test). The error bars correspond to the SEM.

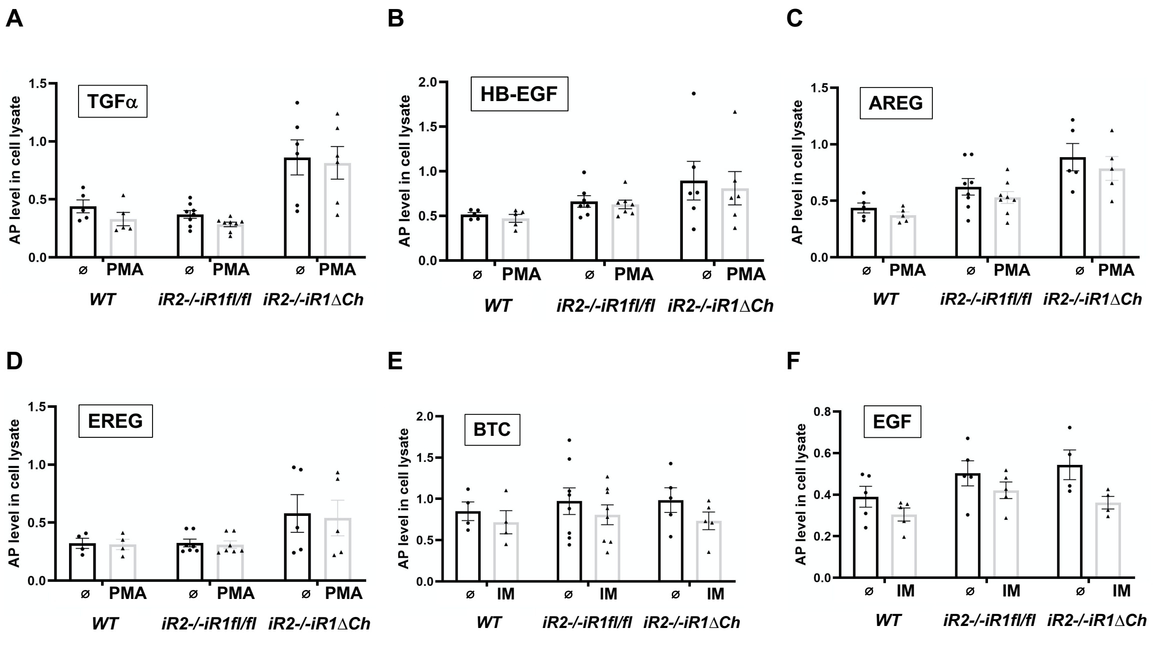

Supplementary Figure 9. Alkaline phosphatase activity in the lysates of primary chondrocytes transfected with AP-tagged EGFR-ligands as an indicator of relative transfection efficiency. The graphs show the AP activity in the cell lysates of the primary chondrocytes used for the shedding experiments in Figure 6 to confirm comparable expression of the cell associated and membrane-anchored AP-tagged EGFR-ligands. In the case of the ADAM17-dependent EGFR-ligands (TGFα, AREG, HB-EGF, EREG), the higher levels in *iR2-/-iR1ΔCh* chondrocytes are a consequence of the strongly reduced shedding of these substrates. Thus, the AP levels in the cell lysates show that the decreased activity of the ADAM17-dependent EGFR-ligands in the supernatant of

the *iR2-/-iR1ΔCh* chondrocytes was not caused by low transfection or expression levels in these cells, but instead, that these substrates presumably accumulated in the cells because they were not shed. The expression of the AP-tagged ADAM10-substrates BTC and EGF is comparable between unstimulated and ionomycin-stimulated (IO) samples of different genotypes.

© 2020 by the authors. Submitted for possible open access publication under the terms and conditions of the Creative Commons Attribution (CC BY) license (http://creativecommons.org/licenses/by/4.0/).
